# Clinically viable myocardial CCTA segmentation for measuring vessel-specific myocardial blood flow from dynamic PET/CCTA hybrid fusion

**DOI:** 10.1186/s41824-021-00122-1

**Published:** 2022-02-15

**Authors:** Marina Piccinelli, Navdeep Dahiya, Jonathon A. Nye, Russell Folks, C. David Cooke, Daya Manatunga, Doyeon Hwang, Jin Chul Paeng, Sang-Geon Cho, Joo Myung Lee, Hee-Seung Bom, Bon-Kwon Koo, Anthony Yezzi, Ernest V. Garcia

**Affiliations:** 1 Department of Radiology and Imaging Sciences, Emory University School of Medicine, 1364 Clifton Rd, NE, Atlanta, GA 30322 USA; 2 Department of Electrical Engineering, Georgia Institute of Technology, Atlanta, GA USA; 3 Department of Internal Medicine and Cardiovascular Center, Seoul National University Hospital, Seoul, Korea; 4 Department of Nuclear Medicine, Seoul National University Hospital, Seoul, Korea; 5 Department of Nuclear Medicine, Chonnam National University, Gwangju, Korea; 6 Samsung Medical Center, Heart Vascular Stroke Institute, Seoul, Korea

**Keywords:** Multimodality image fusion, Absolute myocardial blood flow, Vessel-specific quantification, Cardiac PET, Coronary CTA

## Abstract

**Background:**

Positron emission tomography (PET)-derived LV MBF quantification is usually measured in standard anatomical vascular territories potentially averaging flow from normally perfused tissue with those from areas with abnormal flow supply. Previously we reported on an image-based tool to noninvasively measure absolute myocardial blood flow at locations just below individual epicardial vessel to help guide revascularization. The aim of this work is to determine the robustness of vessel-specific flow measurements (MBF^vs^) extracted from the fusion of dynamic PET (dPET) with coronary computed tomography angiography (CCTA) myocardial segmentations, using flow measured from the fusion with CCTA manual segmentation as the reference standard.

**Methods:**

Forty-three patients’ ^13^NH_3_ dPET, CCTA image datasets were used to measure the agreement of the MBF^vs^ profiles after the fusion of dPET data with three CCTA anatomical models: (1) a manual model, (2) a fully automated segmented model and (3) a corrected model, where major inaccuracies in the automated segmentation were briefly edited. Pairwise accuracy of the normality/abnormality agreement of flow values along differently extracted vessels was determined by comparing, on a point-by-point basis, each vessel’s flow to corresponding vessels’ normal limits using Dice coefficients (DC) as the metric.

**Results:**

Of the 43 patients CCTA fully automated mask models, 27 patients’ borders required manual correction before dPET/CCTA image fusion, but this editing process was brief (2–3 min) allowing a 100% success rate of extracting MBF^vs^ in clinically acceptable times. In total, 124 vessels were analyzed after dPET fusion with the manual and corrected CCTA mask models yielding 2225 stress and 2122 rest flow values. Forty-seven vessels were analyzed after fusion with the fully automatic masks producing 840 stress and 825 rest flow samples. All DC coefficients computed globally or by territory were ≥ 0.93. No statistical differences were found in the normal/abnormal flow classifications between manual and corrected or manual and fully automated CCTA masks.

**Conclusion:**

Fully automated and manually corrected myocardial CCTA segmentation provides anatomical masks in clinically acceptable times for vessel-specific myocardial blood flow measurements using dynamic PET/CCTA image fusion which are not significantly different in flow accuracy and within clinically acceptable processing times compared to fully manually segmented CCTA myocardial masks.

## Background

Since the development and validation of the Fractional Flow Reserve (FFR) index (Bruyne and Sarma 2008) to invasively assess the hemodynamic significance of coronary lesions and guide revascularization (Bruyne et al. 2012), novel methodologies based on noninvasive imaging techniques have been investigated and proposed to stratify patients with known or suspected coronary artery disease (CAD) and plan the appropriate treatment. We previously proposed a novel technique based on a multimodality image fusion approach of dynamic positron emission tomography (dPET) and coronary CT angiography (CCTA) for the estimation of vessel-specific rest/stress (r/s) myocardial blood flow (MBF) along the three-dimensional (3D) trajectory of coronary vessels (Piccinelli et al. 2020). The rationale behind this methodology is the creation of an image-based tool to noninvasively estimate the r/s absolute MBF and derived indexes, such as myocardial flow reserve (MFR) (Schindler et al. 2014) and relative flow reserve (RFR) (Lee et al. 2016), at precise locations underneath the perfusing vessel of interest and use it to guide treatment similarly to the invasive assessment of FFR during LV catheterization. The clinical implementation of this approach has potential for the improved localization of culprit lesions by increasing the mismatch between normal and abnormal flow measurements otherwise averaged when using results extracted from standard anatomical vascular territories.

Requirements for the clinical use of our approach include the multimodality image fusion of the dPET data to CCTA-derived anatomy (i.e., the model of the biventricular myocardium including left (LV) and right (RV) ventricles) and our ability to efficiently extract such anatomical information without time-consuming manual processing. An automated technique for the segmentation of the biventricular epicardium (EPI), LV and RV profiles from CCTA images was recently published (Dahiya et al. 2019). A preliminary and thorough validation on the accuracy and robustness of measuring biventricular chambers size and myocardial mass was also recently reported (Piccinelli et al. 2021) with promising results on both aspects, particularly on its clinical feasibility. However, these studies did not determine the influence of the extracted anatomy on the fusion procedure and consequently on the accuracy of our vessel-specific flow (MBF^vs^) estimates.

The aim of this work was to determine the dependence of MBF^vs^ measurements from the fusion of dPET with *clinically viable* CCTA myocardial segmentation, using as reference standard the MBF^vs^ obtained from fusion of dPET with the manually delineated myocardium. Clinically viable segmentation was defined as fully automated segmentation of LV, RV and EPI borders followed by brief manual border editing in contrast to the two hours usually required for a complete manual border definition of the myocardium that could cover up to 250 CCTA slices.

The agreement between the differently obtained MBF^vs^ was assessed using the Dice similarity coefficient (DC) (Dice 1945) and was determined in terms of classification of the resulting flow values in normal versus abnormal according to ranges for MBF^vs^ developed with our methodology from a cohort of patients with low risk of CAD (Piccinelli et al. 2020).

## Methods

Our overall approach to determine the role of the automated CCTA segmentation on MBF values was to measure the accuracy of MBF^vs^ resulting from the fusion of dPET data with three differently extracted anatomical models. These models were as follows: (1) a totally manual (M) model, which also represented our selected standard of reference, (2) a fully automated segmented (FA) model, obtained with our published technique (Dahiya et al. 2019; Piccinelli et al. 2021), and (3) a corrected (C) model, where major inaccuracies in the automated segmentation were identified during quality control assessment of the borders and briefly edited by an expert in cardiovascular anatomy. Inaccuracies to be manually corrected were visually identified as automated contours that markedly deviated from the myocardial surface or the chambers edges. The resulting three sets of r/s MBF^vs^ were compared to previously developed ranges of flow normality along major vessels in the three vascular territories (Piccinelli et al. 2020) and their agreement was measured with a technique based on DC.

### Study population and imaging datasets

Multimodality image datasets collected in the context of the multisite DEMYSTIFY study (AlBadri et al. 2020) (ClinicalTrails.gov registration number NCT04221594) were used for this investigation. Each dataset is comprised of static, gated and dynamic r/s ^13^NH_3_ PET, CCTA and invasive coronary angiograph (ICA). The first 43 cases included in the DEMYSTIFY database were selected for this study. These patient data were contributed by collaborators in S. Korea: Seoul National University Hospital (SNUH, 35 cases), Samsung Medical Center (SMC, 2 cases) and Chonnam National University Hospital (CNUH, 6 cases). The image acquisitions were performed after local IRB committee approval and consent forms were obtained from all subjects.

All centers used equivalent clinical protocols for both cardiac PET and CCTA imaging as previously reported (Piccinelli et al. 2020; AlBadri et al. 2020). Briefly, prior to the cardiac PET, patients were asked to fast overnight, abstain from caffeine-containing beverages and stop vasodilator medications (such as beta-blockers or calcium channel blockers) for 24 h. The imaging protocol consisted of a low-dose CT scan for attenuation and scatter correction, followed by the injection of 370 MBq bolus of ^13^NH_3_ and the resting dynamic PET acquisition. Hyperemia was induced by a 3-min intravenous infusion of adenosine (140 μg/kg/min) followed by a second dose of ^13^NH_3_ and the stress PET acquisition. All images were acquired in list mode and binned according to the following temporal schemes: 12 × 10 s, 6 × 30 s, 2 × 60 s, 1 × 180 s for SNUH, 12 × 5 s, 6 × 10 s, 3 × 20 s, 6 × 30 s for SMC and CNUH. Prospectively ECG-gated contrast-enhanced CT images were acquired following standard clinical guidelines after the injection of 60 mL of nonionic contrast agent at 4 mL/s. Sublingual nitroglycerine was administered to all patients to facilitate visualization of coronary vessels. The diastolic phase (usually located between 65 and 85% of the cardiac cycle) was selected for the extraction of the anatomy as it allowed a relative motion-free visualization of the major vessels and the myocardium. Coronary CTA images were acquired either before or after cardiac PET. Image acquisition was performed after local IRB committee approval and consent forms were obtained from all subjects. All images were saved in DICOM format, anonymized and securely transferred to the core laboratory at Emory University for subsequent processing.

### CCTA-derived anatomy extraction

A requirement of our approach for the calculation of MBF along coronary 3D paths is the extraction of the anatomical information from the CCTA images, specifically binary masks identifying LV, RV and EPI, which are used for the 3D image fusion of dPET and CCTA images, and coronary centerlines to localize the myocardium underneath the vessels. The extraction of the anatomical masks is a particularly challenging task. If manually performed, it can require up to two hours work by a trained user. Fully automated segmentations may require a quality control assessment, but they guarantee time efficiency and clinical feasibility. The technical details of our automated segmentation algorithms are presented elsewhere, as well as an initial validation of their robustness when compared to manual segmentation used as the reference standard (Dahiya et al. 2019; Piccinelli et al. 2021).

**Fig. 1:**
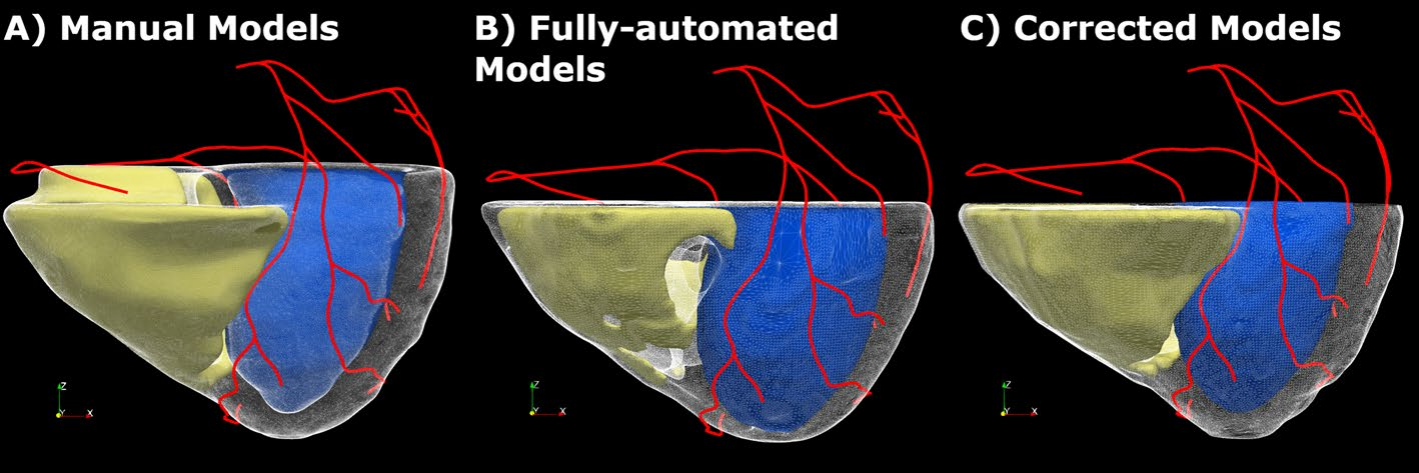
3D models reconstructed from the CCTA-derived anatomical binary masks of LV, RV and EPI obtained by manual delineation (**A**), by means of a fully automated segmentation algorithm (**B**) and by manual correction of the automatically obtained masks (**C**). CCTA-derived coronaries centerlines also shown on all models. *LV* left ventricle, *RV* right ventricle, *EPI* biventricular epicardium

After re-orientation of the transaxial CCTA images in the short-axis (SA) direction, an isotropic volume of 512 × 512 × 512 pixels was created with the myocardium encompassing between 200 and 250 slices. A resampled version of this high-resolution dataset was created with a spacing 4 × the native planar one and used as input to the automated algorithms. M masks were obtained by manual delineation of the myocardium and blood pools on the high-resolution volume by an expert in cardiac imaging using an in-house developed software for editing myocardial borders. FA masks were generated with our proposed automated algorithms (Dahiya et al. 2019) from the low-resolution version of the SA-oriented CT images. Finally, FA masks were reviewed and C masks obtained as the result of a limited manual correction step. The manual corrections had the final goal of ensuring the completion of the fusion procedure and specific guidelines were provided to perform such corrections. Since the image registration (as described in the next section) relied on a univocal identification of the ventricles and myocardium, specific features of the segmented heart morphology (e.g., a clear separation between epicardium and endocardium along the septum and at the apex) were targeted. Only additional major segmentation errors (e.g., heavy RV encroachment into the liver space) were manually corrected, but minor ones ignored. The aim of this investigation was not to measure the performance of our automated segmentation tools, but to determine whether low-resolved automated or semi-automated segmented masks obtained in time-efficient fashion could guide the fusion phase in the context of our proposed method for MBF^vs^ quantification and lesion significance assessment. Efforts were made to minimize user interactions in order to truly assess the MBF^vs^ calculation when guided by FA masks and mimic clinical environment settings where brief high-level user guidance in automated medical image processing tasks is common. LV, RV and EPI borders were reviewed slice-by-slice and minimally corrected where/if necessary. The time devoted to these corrections was recorded for each case. Finally, coronary centerlines were manually extracted with in-house developed image processing tools (VMTK 2021) from CCTA images. Figure 1 shows the 3D biventricular models with coronary centerlines obtained from the 3 sets of anatomical masks.

### PET/CCTA image fusion, flow model and vessel-specific MBF (MBF^vs^) calculation

**Fig. 2:**
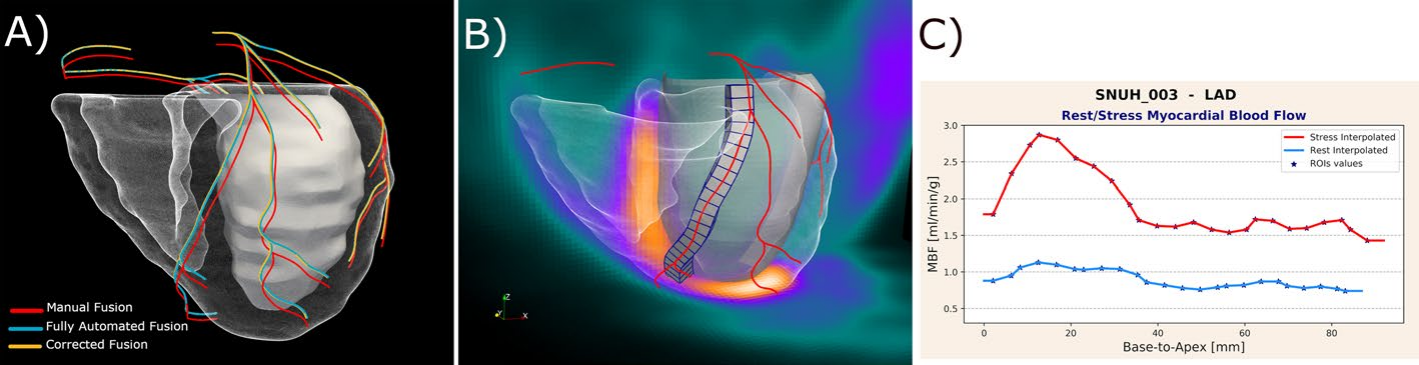
**A** 3D display of CCTA-derived coronaries centerlines after fusion of dPET data with CCTA-derived anatomical masks obtained by manual delineation (red), with a fully automated segmentation algorithm (blue) and after manual revision of the automatically obtained masks (orange). **B** Extraction of the myocardium subtended to the LAD centerline and its discretization in ROIs from which TACs will be derived and used in the model for MBF quantification. **C** Derived 2D plots of stress (red) and rest (blue) MBF^vs^ along the LAD length in mm from the base to the apex of the heart; markers * indicate the flow values obtained from the cubic ROIs; continuous curves obtained by interpolation. *LAD* left anterior descending artery, *TACs* time activity curves, *ROI* region of interest

As previously described (Piccinelli et al. 2020), our methodology to calculate flow along vessel centerlines relies on the fusion of dPET and anatomy. Importantly, the dPET sequence was first corrected for inter-frame motion (Nye et al. 2021) and the resulting images were used for the remainder of the processing. The registration procedure consisted of the following steps (performed for rest and stress separately): (1) a summed PET image (PET_sum_) was created from the dynamic frames of the second half of the acquisition interval; (2) the LVs segmented from the CCTA and PET_sum_ were rigidly registered; and (3) mutual information-based techniques were used to align PET_sum_ with the CCTA-derived biventricular mask using LV and RV structures from both modalities. More detailed information can be found in previous works (Faber et al. 2011; Piccinelli et al. 2018). The same transformations were finally applied to align the coronary centerlines to the PET space. Using the registered centerline as guidance, the myocardium subtended to each vessel was identified on the dPET images and discretized in contiguous cubic volumetric elements with a longitudinal size of 4 mm that followed the 3D trajectories. The MBF quantification was performed using standard tracer kinetic modeling techniques. Time-varying tracer concentrations were extracted in the form of time activity curves (TAC) from specific regions of interests (ROIs), namely vascular territories and the arterial blood, and used as input data into a 2-tissue compartmental model (Hutchins et al. 1990) that returned r/s MBF values for each identified ROI. The TACs from the contiguous cubic ROIs representing the myocardium subtended to vessels were also fed to the model, thus generating r/s flow profiles along the vessel (MBF^vs^). As the fusion procedure was performed with M, FA and C anatomical masks, 3 sets of r/s MBF^vs^ curves were extracted for each analyzed vessel (respectively, ${\text{MBF}}_{{\text{M}}}^{{{\text{vs}}}}$, ${\text{MBF}}_{{{\text{FA}}}}^{{{\text{vs}}}}$ and ${\text{MBF}}_{{\text{C}}}^{{{\text{vs}}}}$). Figure 2 shows the crucial processing steps for one of the analyzed cases: (A) the 3 sets of post-fusion coronary centerlines showing limited differences in orientation and position, (B) the extraction of the myocardium subtended to a vessel and its discretization in ROIs, and (C) the left anterior descending (LAD) r/s ${\text{MBF}}_{{\text{M}}}^{{{\text{vs}}}}$ presented as a two-dimensional (2D) graph of flow values along the vessel length (in mm, from the base to the apex of the heart). The actual flow values obtained from the cubic ROIs are indicated by the markers.

**Fig. 3:**
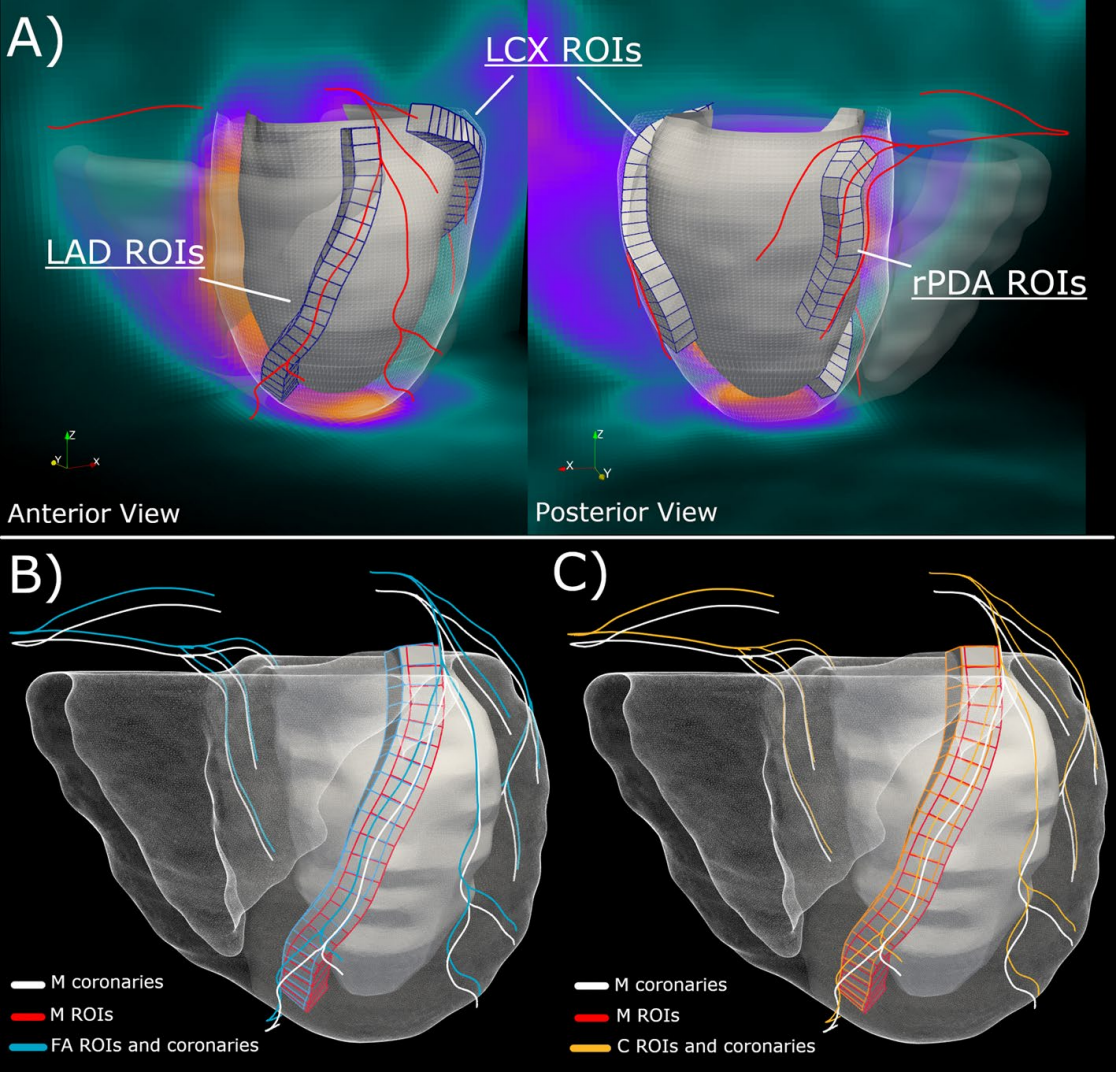
**A** Anterior and posterior views of the heart 3D anatomy showing the extraction of the vessel-specific myocardium subtended to the LAD, LCX and rPDA and their discretization in ROIs for dPET sampling and TAC extraction. **B** Comparison between the manual LAD vessel-specific ROIs (red) and the fully automated ROIs (blue) with their corresponding centerlines. **C** same as in B comparing manual (red) and corrected (orange) LAD ROIs. *M* manual, *FA* fully automated. **C** corrected. *ROI* region of interest, *LAD* left anterior descending artery, *LCX* left circumflex artery, *rPDA* right posterior descending artery, *TACs* time activity curves

For the current investigation, r/s MBF^vs^ profiles were extracted for one vessel per vascular territory for each analyzed case: LAD, one major vessel from the lateral wall (indicated as LCX), and the right posterior descending artery (rPDA). Figure 3A shows the anterior and posterior views of the heart with the vessel-specific modeling to probe three vessels for one of the analyzed cases. Figure 3B, C illustrates the differences introduced by the fusion of FA and C models with respect to the reference standard (M) in terms of LAD vessel-specific ROI spatial location.

### Normal/abnormal classification of MBF^vs^ curves

**Fig. 4:**
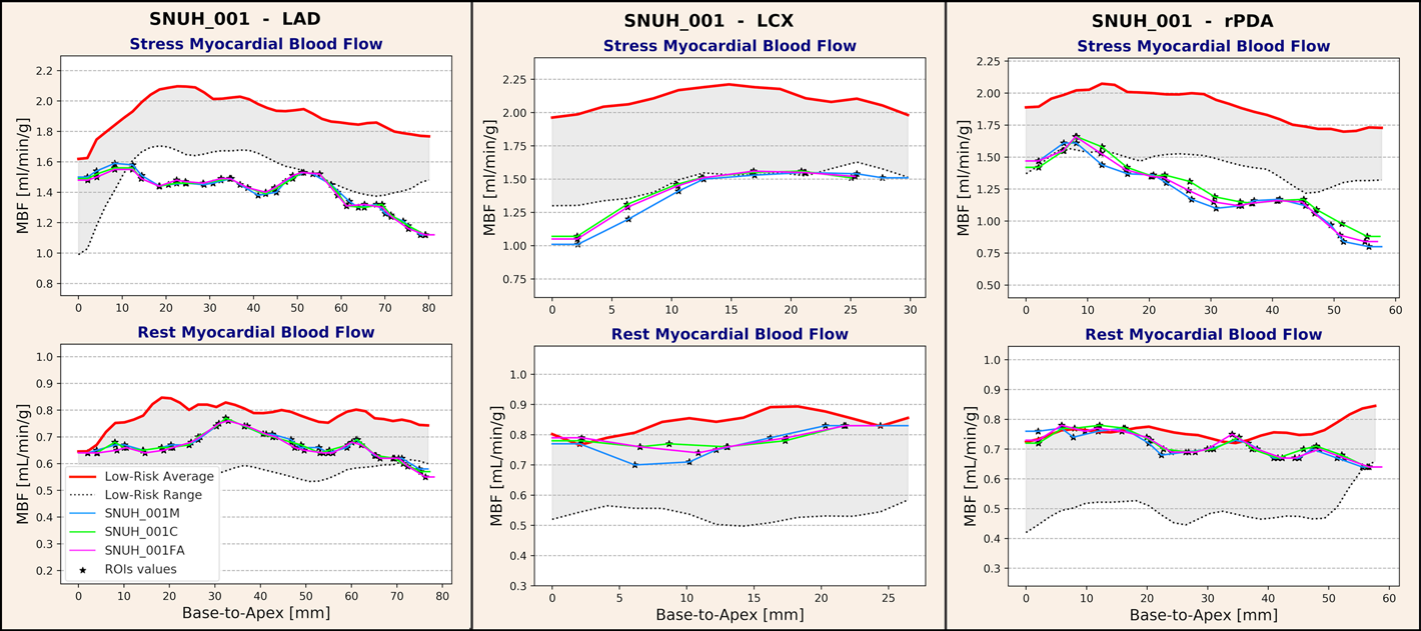
2D plots of stress, rest ${\text{MBF}}_{{{\text{FA}}}}^{{{\text{vs}}}}$ (magenta), ${\text{MBF}}_{{\text{C}}}^{{{\text{vs}}}}$ (green) and ${\text{MBF}}_{{\text{M}}}^{{{\text{vs}}}}$ (blue) from the base to the apex in mm for the LAD, LCX and rPDA of one of the analyzed cases. The flow values profiles are compared to previously developed r/s MBF^vs^ ranges for patients with low risk of CAD (grayed area). Vessel-specific ROI values indicated with markers (*); continuous curves obtained by interpolation. For the displayed case, stress (rest) FA Dice coefficients were for LAD, LCX and rPDA, respectively, 0.96, 0.86 and 0.94 (0.96, 1.00 and 0.94); and stress (rest) C Dice coefficients were, respectively, 0.96, 0.86 and 0.96 (0.96, 1.0 and 1.0). *ROI* region of interest, *LAD* left anterior descending artery, *LCX* left circumflex artery, *rPDA* right posterior descending artery

The MBF^vs^ methodology was previously applied to define ranges of r/s MBF^vs^ along the major vessels supplying the LV based on a cohort of patients (*n* = 15) with low risk of CAD who underwent ^13^NH_3_ PET and CCTA (Piccinelli et al. 2020). Since the final goal of this approach is to determine whether indexes based on MBF become abnormal at specific locations along a vessel, thus indicating the presence of flow-limiting lesions that require revascularization, these low-risk ranges were used to prospectively stratify MBF^vs^ into normal (within the range) versus abnormal (below the range). In the context of this investigation, the low-risk ranges were used to determine whether the three sets of r/s MBF^vs^ curves were classified in the same way and/or to what extent the automated and the corrected curves differed from the ones obtained when the manually delineated anatomy was used for image registration. Figure 4 displays 2D plots of the low-risk ranges and r/s ${\text{MBF}}_{{\text{M,FA,C}}}^{{{\text{vs}}}}$ for the three vessels considered for one of the analyzed cases.

To quantify the discrepancies in the normal/abnormal classification between ${\text{MBF}}_{{{\text{FA}}}}^{{{\text{vs}}}}$ and ${\text{MBF}}_{{\text{C}}}^{{{\text{vs}}}}$ compared to ${\text{MBF}}_{{\text{M}}}^{{{\text{vs}}}}$ curves, the Dice coefficient (Dice 1945) (DC) was calculated. DC measures spatial overlapping commonly used to rate the performance of segmentation algorithms with values between 0 (no overlapping) to 1 (complete overlapping). Adapted to our analysis DC_FA_ was computed to quantify the number of flow values (i.e., ROI values) with matched classification between ${\text{MBF}}_{{{\text{FA}}}}^{{{\text{vs}}}}$ and ${\text{MBF}}_{{\text{M}}}^{{{\text{vs}}}}$ based on low-risk ranges. Analogously, DC_C_ was derived from the comparison of flow values classification between ${\text{MBF}}_{{\text{C}}}^{{{\text{vs}}}}$ and ${\text{MBF}}_{{\text{M}}}^{{{\text{vs}}}}$. DC_FA_ and DC_C_ were computed globally by pooling all the ROI samples from all the vessels. A parallel analysis was conducted per vascular territory as image quantification performance may differ among vascular territories.

### Inter-user variability analysis

To assess the impact of the user corrections of the anatomical masks on the fusion procedure and consequently on the MBF^vs^ profiles, a second user was tasked with correcting the automatically extracted anatomy in a subset of the analyzed cases. The second user had a similar background on cardiac anatomy and was provided with the same guidelines on how to perform the corrections. The cases were selected to represent different degree of agreement between ${\text{MBF}}_{{\text{C}}}^{{{\text{vs}}}}$ and ${\text{MBF}}_{{\text{M}}}^{{{\text{vs}}}}$. The DC_C_ obtained after the two manual correction phases (indicated as ${\text{DC}}_{{\text{C}}}^{1}$ and ${\text{DC}}_{{\text{C}}}^{2}$) were calculated for a total of 12 vessels, three vessel per case.

### Statistical analysis

Continuous variables are presented as mean ± SD, while discrete variables are expressed as frequency distributions and percentages. The DC analysis was performed for all pooled vessels and separated by vascular territory and presented as mean values for each group. The presence of statistically significant differences in classification agreements between segmentation modes was tested by way of a Student’s *t* test, with *p* < 0.05 as level of significance. The Student’s *t* test was also used to determine whether the two manual corrections of the anatomical masks provided significantly different classifications with respect to the normal/abnormal ranges.

## Results

### Anatomical masks creation

Forty-three consecutive patients collected from the imaging database of the DEMYSTIFY study were analyzed and processed for this investigation. Basic demographic characteristics and clinical data relevant for this investigation are summarized in Table 1. The cohort contained a mix of patients with no physiologically significant CAD and patients with various degree of disease (as determined by invasive FFR index detailed in Table 1) to assess agreement in flow values in a variety of clinical scenarios. For each case M, FA and C anatomical masks (representing LV, RV and EPI) were defined from CCTA images (Fig. 1). The manual segmentation of the myocardium was time-intensive, ranging 1.5–2 h work per case. The fully automated segmentation was accomplished on average in 15 s per image, which confirms the clinical feasibility of the technique (Piccinelli et al. 2021). FA masks corrections required a more variable time, depending on the quality of the automated segmentation results, as detailed below. Three centerlines following LAD, LCX and rPDA were manually delineated, with processing time that ranged 2–5 min per case. The three autonomous r/s image registration procedures were performed, the coronary centerlines accordingly fused to the dPET data and the MBF^vs^ extracted. With the exclusion of the anatomical information extraction (myocardium and centerlines), the successive processing was fully automated and ranged 2–3 min per vessel.

**Table 1:** Demographic and clinical data for patients’ population

Patient demographic	All	M	F
*n*	43	37	6
Age	64	64	67

CAD risk factor	*n* (%)		
Hypertension	31 (72%)		
Diabetes	13 (30%)		
Hyperlipidemia	38 (88%)		
Tobacco use	24 (56%)		
Early CAD family history	4 (9%)		
Prior stents	25 (58%)		
Prior MI	4 (9%)		
CABG	0 (0%)		
Heart failure	0 (0%)		

Vessels examined	FFR < 0.8	FFR ≥ 0.8	No FFR
*n* = 124			
LAD	14	26	2
LCX	4	20	18
rPDA	3	24	13

### PET/CCTA fusion

Rest/stress image fusion was carried out for all cases with three sets of anatomical masks. In 37% of the 43 cases, no border corrections were necessary to complete the fusion, while editing was required for the remaining cases. As for their extent, the corrections were as follows: 1—border correction for 14/43 (33%), 2—border correction for 16/43 (37%) and 3—border correction for 7/43 (16%). The most common corrections were on the biventricular border (65%), followed by the RV (53%) and the LV profiles (35%). The most common issue related to the FA segmentations was the inadequate coverage at the apex affecting only few slices of the total myocardial coverage; minimal adjustments were required for these cases which were achieved in 2–3 min on average. About 50% of the cases required such brief interventions which fell within the provided guidelines for manual corrections of the FA masks to create C masks. Situations with overlapping epicardium and endocardium (commonly present at the septum) required the simultaneous modification of two profiles, hence approximately twice the correction time.

### MBFVS calculation and accuracy

**Fig. 5:**
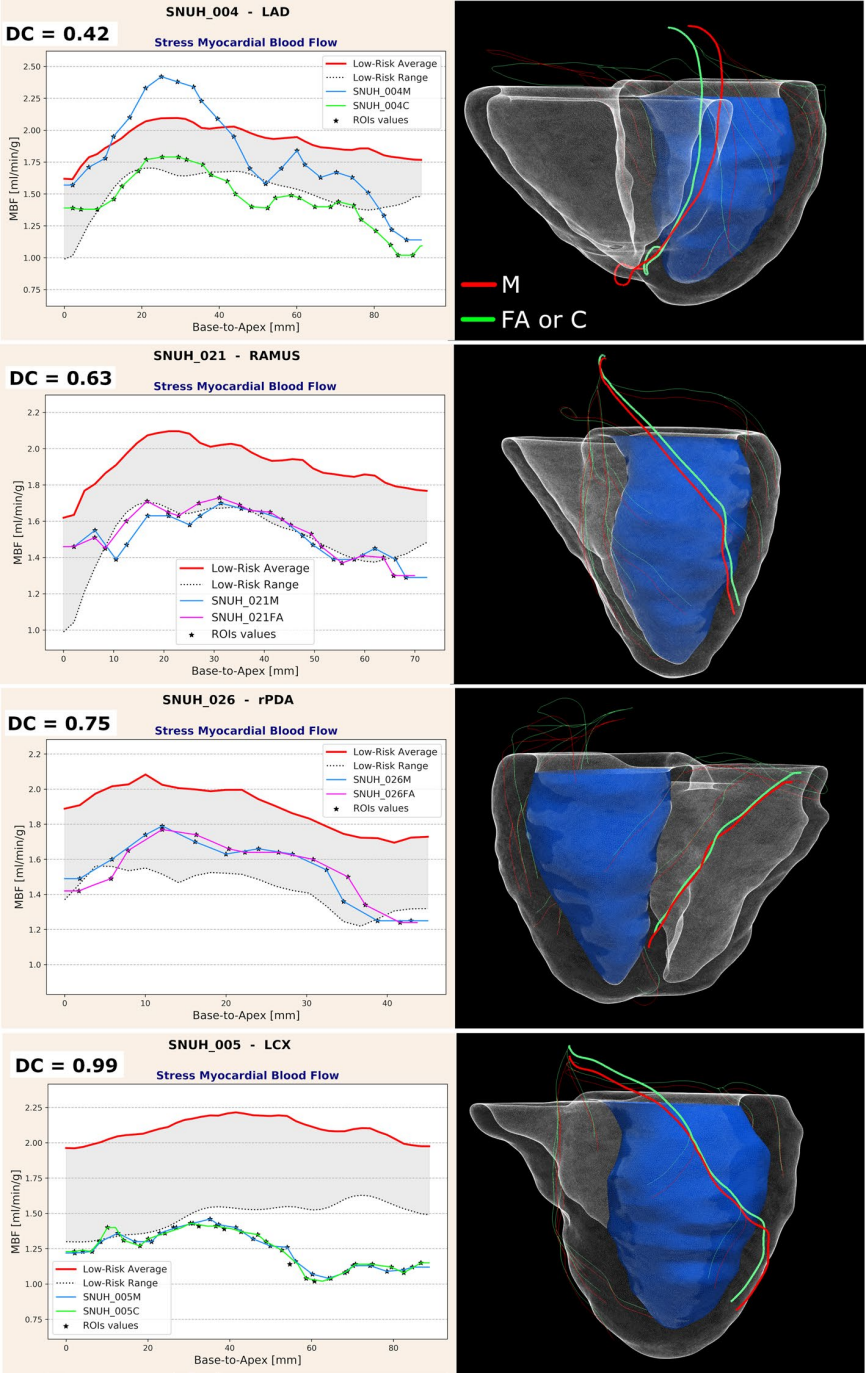
Dice coefficient (DC) values for a series of analyzed vessels selected to show increased DC accuracy of ${\text{MBF}}_{{{\text{FA}}}}^{{{\text{vs}}}}$ or ${\text{MBF}}_{{\text{C}}}^{{{\text{vs}}}}$ when compared to manual ${\text{MBF}}_{{\text{M}}}^{{{\text{vs}}}}$. Figure displays 2D MBF profiles and correspondent 3D anatomies; thicker centerlines represent the analyzed vessel: Red centerlines are obtained from fusion with manual (M) anatomical masks and green centerlines from fusion with either fully automated (FA) or corrected (C) masks

LAD, LCX and rPDA ${\text{MBF}}_{{\text{M,FA,C}}}^{{{\text{vs}}}}$ were calculated for all cases that could be directly fused: 43 cases with M masks, 43 cases with C masks and 16 cases with intact FA masks. In three cases, no vessel suitable for analysis could be found in the LCX territory, and in 2 cases, the rPDA could not be identified from the CCTA images. A total number of 124 vessels were analyzed after fusion with the M and C masks yielding 2225 stress and 2122 rest flow values. Forty-seven vessels were analyzed after fusion with the FA masks producing 840 stress and 825 rest flow samples. Global and vessel-based DC were computed, and results are reported in Table 2. Figure 4 shows an example of good agreements between ${\text{MBF}}_{{\text{FA,C}}}^{{{\text{vs}}}}$ and ${\text{MBF}}_{{\text{M}}}^{{{\text{vs}}}}$, and Fig. 5 displays cases with increasing DC values, from the worst case (DC = 0.42) to instances of improved accuracy. 3D models and coronary centerlines are also displayed to assess the corresponding fusion results.

**Table 2:** Agreement between ${\text{MBF}}_{{{\text{FA}}}}^{{{\text{vs}}}}$ and ${\text{MBF}}_{{\text{C}}}^{{{\text{vs}}}}$ compared to ${\text{MBF}}_{{\text{M}}}^{{{\text{vs}}}}$ measured by way of the Dice coefficient

	Manual versus fully automated masks		Manual versus corrected masks	
	Stress MBF	Rest MBF	Stress MBF	Rest MBF
*N* samples	840	825	2225	2122
Global Dice coefficient	0.95	0.97	0.94	0.98
LAD Dice coefficient	0.96	0.96	0.95	0.98
LCX Dice coefficient	0.94	0.96	0.95	0.99
rPDA Dice coefficient	0.95	0.98	0.93	0.98

No statistically significant differences were found between global versus per territory assessments, between rest and stress MBF quantifications or due to the anatomical masks used to perform dPET/CCTA fusion

When analyzed on a case-by-case basis after fusion with C masks, 63% and 85% of the vessels had perfectly matched ${\text{MBF}}_{{\text{C}}}^{{{\text{vs}}}}$ values (i.e., DC = 1), respectively, for stress and rest, while 80% and 94% had stress and rest DC_C_ ≥ 0.9, respectively. When FA masks were used, 62% and 72% of the vessels had perfectly matched stress and rest ${\text{MBF}}_{{{\text{FA}}}}^{{{\text{vs}}}}$, while 83% and 94% had stress and rest DC_FA_ ≥ 0.9. No statistical differences were found in the normal/abnormal classifications between manual and corrected or manual and fully automated, both globally and per vessel. When the 16 cases fused with native FA masks and C masks were analyzed, no statistical differences could be found in the respective classification results.

### Inter-user variability analysis

The 4 cases displayed in Fig. 5, which exhibited a wide range of agreements between the different MBF^vs^ profiles and included the case with the worst performance in terms of fusion accuracy and consequently of MBF^vs^ assessment, were used for the inter-user variability analysis. After manual corrections from the two users, MBF^vs^ profiles were extracted along the LAD, LCX and rPDA of each case, which accounted for 223 stress and 218 rest flow values. Global ${\text{DC}}_{{\text{C}}}^{1}$ and ${\text{DC}}_{{\text{C}}}^{2}$ were, respectively, 0.86 and 0.85 at stress and 0.97 and 0.99 at rest. When vessels were separated by vascular territory ${\text{DC}}_{{\text{C}}}^{1}$ and ${\text{DC}}_{{\text{C}}}^{2}$ were for the LAD territory, respectively, 0.83 and 0.82 at stress, 0.97 and 1.00 at rest; for the LCX territory 0.89 and 0.84 at stress, 0.93 and 0.95 at rest; and for the RCA territory 0.87 and 0.94 at stress, 1.00 and 1.00 at rest. No statistically significant differences were found in the classification of individual vessels.

## Discussion

In previous publications (Piccinelli et al. 2020; AlBadri et al. 2020), we presented a novel methodology to quantify MBF along vessel-specific 3D trajectories by fusing CCTA images to dPET. This multimodality image approach requires the efficient extraction of the heart anatomy from the CCTA acquisitions, namely blood pools and biventricular epicardium. In this work, we evaluated the ability of two sets of anatomical masks to guide efficiently and accurately image fusion and MBF^vs^ extraction and permit its classification in normal versus abnormal values: the fully automated masks obtained with our previously developed algorithms for automated CCTA image segmentation (Dahiya et al. 2019; Piccinelli et al. 2021) and the corrected masks obtained after manual editing of the automated results by an expert user. The normal/abnormal classification of flow values was performed by comparing the calculated profiles to normality ranges previously developed from a cohort of subjects with low risk of CAD (Piccinelli et al. 2020). Our results indicate that both the automated (FA) and semi-automated (C) masks allow a highly accurate extraction of MBF^vs^ along vessel trajectory that matched the values obtained when standard of reference manual segmentations were used (Table 2). The processing time from original images to MBF^vs^ displays for the complete fully automated procedure was on average 5–7 min, compatible with the application of the methodology in a clinical environment. We determined that, although a majority of the fully automated borders required manual correction before dPET/CCTA image fusion, this editing process was brief (2–3 min) allowing a 100% success rate of extracting MBF^vs^. Importantly, these MBF^vs^ were shown to accurately categorize normal/abnormal flow along the major vessel path for both rest and stress flows. Moreover, for those cases that were processed with intact FA masks and C masks, no significant differences were found in the normal/abnormal classification of ${\text{MBF}}_{{{\text{FA}}}}^{{{\text{vs}}}}$ and ${\text{MBF}}_{{\text{C}}}^{{{\text{vs}}}}$, substantiating our argument that our algorithms for automated segmentation of CCTA can correctly guide multimodality image fusion, notwithstanding the need for further improvements. Finally, by means of our small inter-user variability analysis, we showed that classification of flow values in normal/abnormal is unlikely to depend upon the user’s limited and guided manual corrections of the anatomical masks.

The use of Dice coefficient to quantify the agreement between the different sets of MBF curves base to apex has also proved reliable. DC has commonly been used as an index of spatial overlapping specifically designed to grade the performance of different segmentation/classification methods. Since our interest is in establishing whether MBF becomes abnormal at specific locations along a vessel 3D trajectory, our proposed methodology can still be described as a classification technique, but with a different tolerance to pixel-level errors in the CCTA segmentations. Determining such a tolerance was the rationale for this work, and our results indicate that even if M, FA and C masks may exhibit differences, the PET coarser image resolution and the discretization of the myocardium subtended to the vessel of interest into ROIs shield MBF values from high variations and consequently major errors in flow assessment. Our implementation of the DC index was also relatively strict in rating the agreement between the different sets of MBF^vs^ curves. The classification was overtly wrong only for a few vessels (3 vessels from 2 cases) and was characterized by DC < 0.5. But as depicted in the series of 2D plots in Fig. 5, even cases with MBF profiles that closely follows the standard of reference ones, the computed DC falls into a low-value range (0.6–0.75). Since our global DC_FA,C_ values are consistently ≥ 0.93, these considerations further strengthen our conclusions on the technical robustness of the approach.

Gould et al. (Gould et al. 2000) introduced the concept of measuring relative tracer uptake base to apex on different LV quadrants and evidenced progressive abnormalities in uptake when considering different patients’ populations from normal subjects to patients with diffuse disease, to patients with overt perfusion defects. Other groups (Valenta et al. 2014, 2017; Bom et al. 2019) have suggested measuring relative perfusion or absolute blood flow “longitudinally” with mixed results. With our approach we aim at overcoming the limitations related to the subdivision of the myocardium in standard territories by closely following 3D coronary paths irrespective of segments location and keeping the myocardial tissue probed to measure flow to a minimum in order to identify significant lesions.

Multimodal image approaches are powerful image-based diagnostic strategies. Yet numerous complicating factors have delayed the development of software tools that implement not just the visualization of synchronized images, but also a comprehensive and synergistic image quantification with extraction of indexes characterizing the anatomy and the function of the organ or the system under study. This work originated in our need to test the technical feasibility and quantify the robustness of our multimodal approach—provided the current state of development of our image processing tools—but it also lays the foundations for the clinical validation of our MBF^vs^ methodology and its potential in identifying flow-limiting lesions and guide revascularization.

### Limitations

While we consider the results presented in this work very promising from the point of view of the clinical feasibility of our proposed method for MBF^vs^ quantification, we recognize the need for additional steps toward its full automation. Further developments of our image segmentation algorithms are currently ongoing with a strong emphasis on the mitigation of the issues requiring manual corrections (i.e., incorrect myocardial thickness at the apex and portions of the septum). One additional limitation also related to the anatomy extraction is that vessel centerlines are currently manually delineated. The required processing is usually simple and quick to complete and as such, has not been the focus of our developments in automation. Nevertheless, numerous techniques exist for semi-automated retrieval of coronary centerlines to further make the methodology accessible and applicable (Li and Yezzi 2007) and will be investigated in future works.

## Conclusions

Fully automated and manually corrected myocardial CCTA segmentation provide anatomical masks for vessel-specific myocardial blood flow measurements using dynamic PET/CCTA image fusion which are not significantly different in flow accuracy and within clinically acceptable processing times compared to fully manually segmented CCTA myocardial masks.

## Data Availability

The datasets generated and/or analyzed in the current study are not publicly available but may be available from the corresponding author on reasonable request. The imaging data are currently being collected as part of the funded NIH R01 grant. Original imaging data and results will be made publicly available at the end of grant.

## Abbreviations

CAD: Coronary artery disease
PET: Positron emission tomography
CCTA: Coronary computed tomographic angiography
MBF: Myocardial blood flow
DC: Dice similarity coefficient
LV: Left ventricle
RV: Right ventricle
EPI: Epicardium
